# Synthetic bacterial consortia transplantation for the treatment of *Gardnerella vaginalis*-induced bacterial vaginosis in mice

**DOI:** 10.1186/s40168-023-01497-y

**Published:** 2023-03-20

**Authors:** Yunxia Li, Wei Zhu, Yan Jiang, Duncan James Lessing, Weihua Chu

**Affiliations:** 1 Department of Pharmaceutical Microbiology, School of Life Science and Technology, China Pharmaceutical University, Nanjing, 210009 China; 2 Animal, Plant and Food Inspection Center of Nanjing Customs, Nanjing, 210019 China

**Keywords:** Bacterial vaginosis, Lactic acid bacteria, Synthetic bacterial consortia transplantation, Vaginal microbiota

## Abstract

Bacterial vaginosis (BV) is a disease caused by vaginal microbiota dysbiosis. Here, we propose the use of synthetic bacterial consortia transplantation (SBCT) for the treatment of *Gardnerella vaginalis*-induced BV mice. The results showed that SBCT significantly reduced vaginal tissue damage and restored the vaginal microbiota, decreased the secretion of pro-inflammatory cytokines (IL-1β and IL-8), and suppressed NF-κB activation. IL-17, iNOS, and COX-2 expression in vaginal tissue were also down-regulated. However, IL-10 and Foxp3 showed up-regulated expression in mice. Compared with vaginal microbiota transplantation (VMT), results indicated that VMT was more effective than SBCT in suppressing *G. vaginalis*-induced inflammation. The obtained results suggest that synthetic bacterial consortia might be used as a potential biotherapeutic agent for the treatment of *G. vaginalis*-induced bacterial vaginosis.

Video Abstract

**Supplementary Information:**

The online version contains supplementary material available at 10.1186/s40168-023-01497-y.

## Introduction

The healthy vaginal microbiota of women has low diversity but plays an essential role in maintaining health and preventing vaginal infections. When there is a high diversity, this equals dysbiosis [42]. The vaginal microbiota of healthy women is dominated by *Lactobacillus*, including *L. crispatus*, *L. iners*, and *L. gasseri* [4]. Some *Lactobacillus* strains isolated from the vagina of healthy women to protect against *G. vaginalis* by producing hydrogen peroxide, lactic acid, and bacteriocins [1, 9, 19], which ameliorate the adverse effects of harmful microbiota by their anti-inflammatory effect on the vagina and activation of the host immune response [12, 35]. The bacterial composition of the vaginal microbiota differs considerably in women with and without bacterial vaginosis (BV) [21]. BV is characterized by an important increase in the growth of endogenous bacteria, typically *G. vaginalis* and other pathogenic microorganisms, which is usually dominated by *Lactobacillus* switching to microbiota characterized by the presence of anaerobic bacteria [4, 41]. BV-affected women may experience foul-smelling vaginal discharge, as well as experience burning sensations and discomfort in the vaginal area [3, 23]. In addition, BV may increase the risk of reproductive tract infections, pregnancy complications, and sexually transmitted infections [2]. Notably, the effects of other species found in BV-associated microbiota on biofilm formation and its impact in *G. vaginalis* pathogenicity, which may be the main pathogen of BV [14]. The complex interplay between *G. vaginalis* and specific BV-associated species can enhance vaginal desquamation and the eventual formation of clue cells. It is the facilitation of the destruction of the protective mucus layer of the vaginal epithelium by hydrolyzing the sialic acid on the mucosa [18]. This process has been linked with the development of biofilm. In previous clinical studies, antibiotic therapy (oral metronidazole) was the standard treatment. Chieng et al. research showed that bacterial vaginosis patients treated with antibiotics have resulted in a recurrence rate of 50-70% for the clinic control of the BV [7]. However, antibiotic therapy may lead to vaginal candidiasis and resistant infections [5, 34]. Recent clinical reports, it is worth mentioning that probiotics and vaginal microbiota transplantation (VMT) have been shown to be effective in the treatment of BV [4, 29, 32, 47]. While single-strain probiotics are beneficial to the treatment of BV, their effectiveness is not good enough [6, 20, 48]. Vaginal microbiome transplantation (VMT) from healthy donors is a therapeutic alternative for patients suffering from symptomatic, intractable, and recurrent bacterial vaginosis [28, 33]. However, issues such as the transmission of antibiotic resistance genes, like fecal microbiota transplantation (FMT), remain a significant concern for the acceptability of VMT. The safety and reliability of VMT in treatment need to be further investigated and confirmed. In this study, we used synthetic bacterial consortia transplantation as a new strategy for the control of *G. vaginalis*-induced BV.

## Results

### Vaginal infusion of *G. vaginalis* induced bacterial vaginosis

**Fig. 1:**
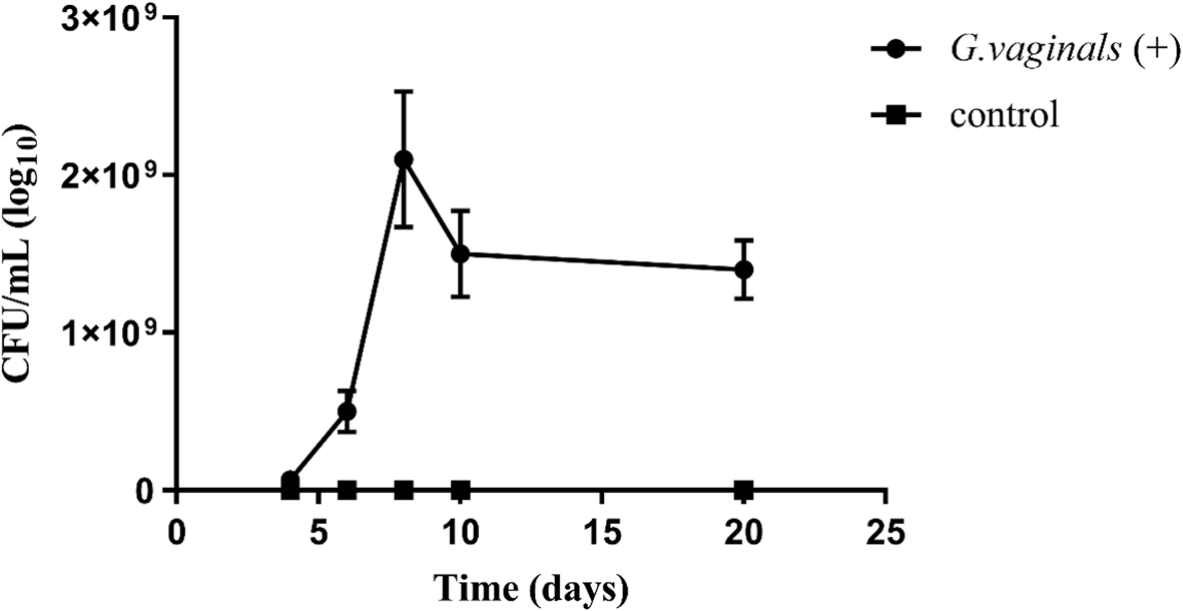
The bacterial burdens in vaginal lavage fluids were assessed longitudinally on days 4, 6, 8, and 21; results are shown as means ± standard error of the mean

To establish *G. vaginalis*-induced BV mice model, the vaginas of female mice were inoculated with 3 × 10^9^ CFU mL^−1^ of *G. vaginalis* for 8 days. After induction of vaginitis, there was no significantly different change in behavior, feces or body weight in the *G. vaginalis* inoculated mice. However, the vaginas of *G. vaginalis* inoculated mice were swollen, reddened and discharged more vaginal secretions when compared to the mice in the normal control group (CON). In the beginning, from the 4th to 8th day post-inoculated with *G. vaginalis*, the total number of *G. vaginalis* in the vaginal discharges of mice increased continuously; and the number of vaginal microbes remained stable from the 10th day post-inoculated with *G. vaginalis*. While no *G. vaginalis* was detected in the CON group (Fig. 1). After induction of vaginitis, the *G. vaginalis*-induced BV mice were divided into three groups, two groups were treated with synthetic bacterial consortia and vaginal microbiota transplantation respectively, and the other group was treated with saline for 2 weeks.

### Histopathological analysis of bacterial vaginosis mice

**Fig. 2:**
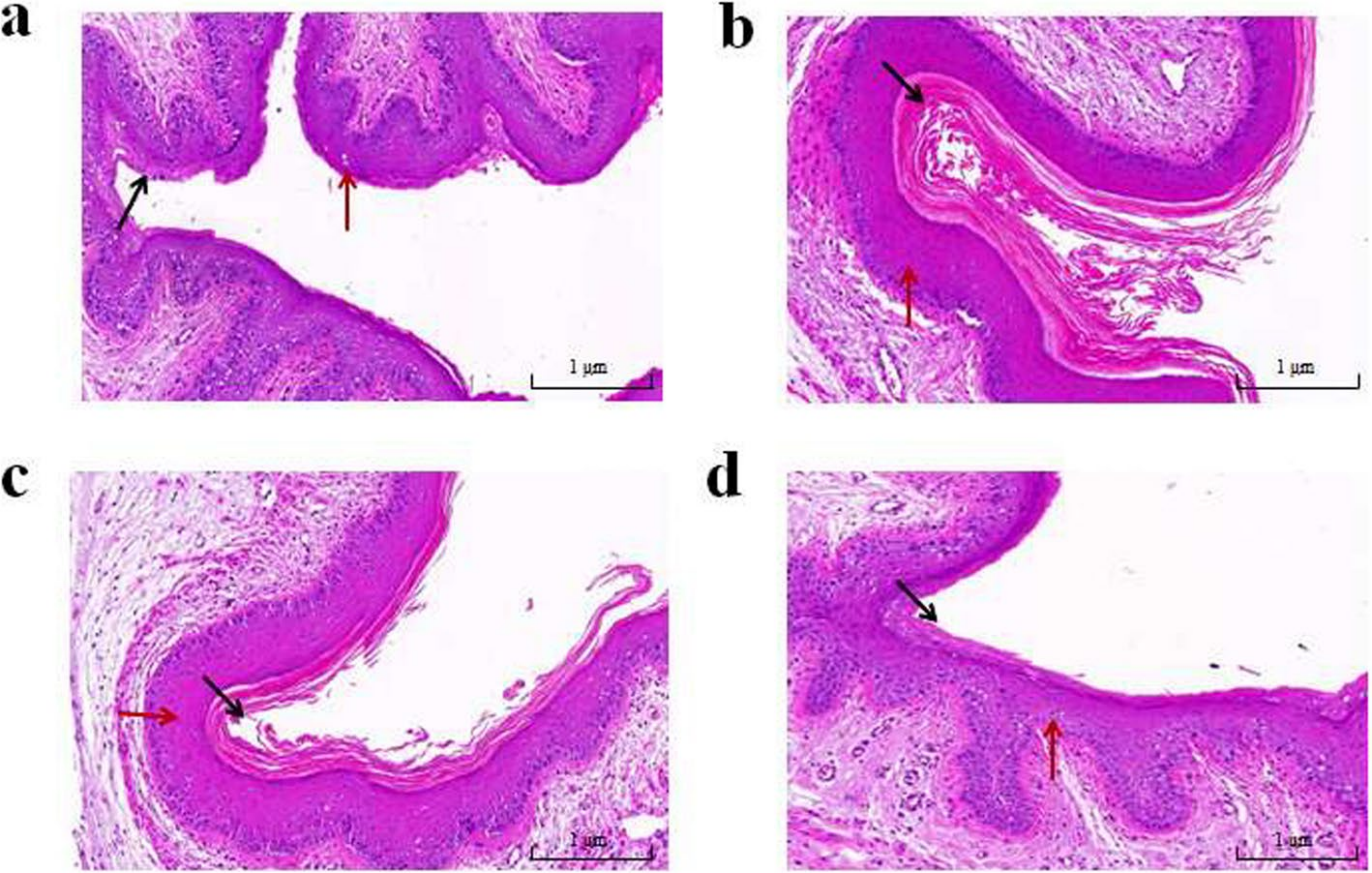
Tissues were stained with hematoxylin-eosin and sections were evaluated by a Pannoramic MIDI digital section scanner. **a** CON, normal control mice. **b** GVI, GV-infected mice. **c** SBCT, synthetic bacterial consortia treatment for mice. **d** VMT, vaginal microbiota transplantation treatment for mice, magnification × 100

We investigated whether synthetic bacterial consortia and vaginal microbiota transplantation treatment attenuate GV-induced vaginitis in mice. Vaginal tissues were filmed and stained with hematoxylin and eosin (H&E) to confirm vaginal tissue damage. The GV-infected group (GVI) of mice had thickened vaginal epithelial tissue (blue arrow) and appeared to become inflammatory cell infiltrated compared to the CON group. In addition, there was a significant increase in the stratification of the epithelium in vaginal tissues. After 2 weeks of synthetic bacterial consortia and vaginal microbiota transplantation treatment, vaginal tissue epithelial mucosa was significantly improved, with reduced stratification (black arrow) and cellular infiltration of the epithelium. Meanwhile, histological examination showed that vaginal tissue treated with VMT had significantly more improved epithelial damage than synthetic bacterial consortia treatment group (SBCT) treated mice compared with CON group (Fig. 2).

### ELISA detection of inflammatory cytokines

**Fig. 3:**
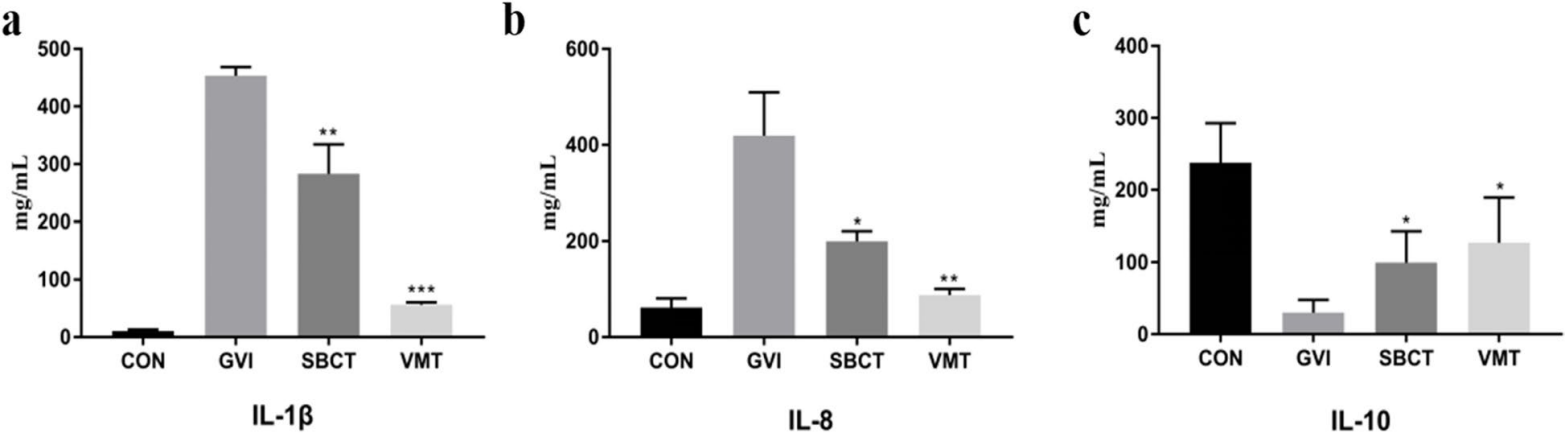
Levels of IL-1β, IL-8, and IL-10 in the serum of mice in different groups. **a** IL-1β, interleukin-1β. **b** IL-8, interleukin-8. **c** IL-10, interleukin-10. Results are shown as means ± standard error of mean. *P < 0.05 vs. GVI group

The immune response to BV is characterized by the release of pro-inflammatory cytokines, mainly interleukin-1β [15]. IL-1β is a key component of the pro-inflammatory cytokine cascade and induces the synthesis of other cytokines, including interleukin-8. IL-8 is associated with elevated epithelial cells and is a pro-inflammatory chemokine that attracts neutrophils to the site of infection. The action of potent immunosuppressive cytokines, IL-10, has a powerful inhibitory effect on T-cell proliferation and inflammation, and it suppresses the production of Th1 cytokines [43]. Therefore, we measured the levels of these cytokines in serum. As shown in Fig. 3, the level of IL-1β and IL-8 increased significantly in the GVI group, but the level of IL-10 decreased. Treatment with synthetic bacterial consortia or vaginal microbiota transplanted can inhibit IL-1β and IL-8 expression in *G. vaginalis*-induced BV mice. In contrast, IL-10 expression was increased in the treatment groups.

### Influence of SBCT and VMT on activation of pro-inflammatory biomarkers in vaginal tissues

**Fig. 4:**
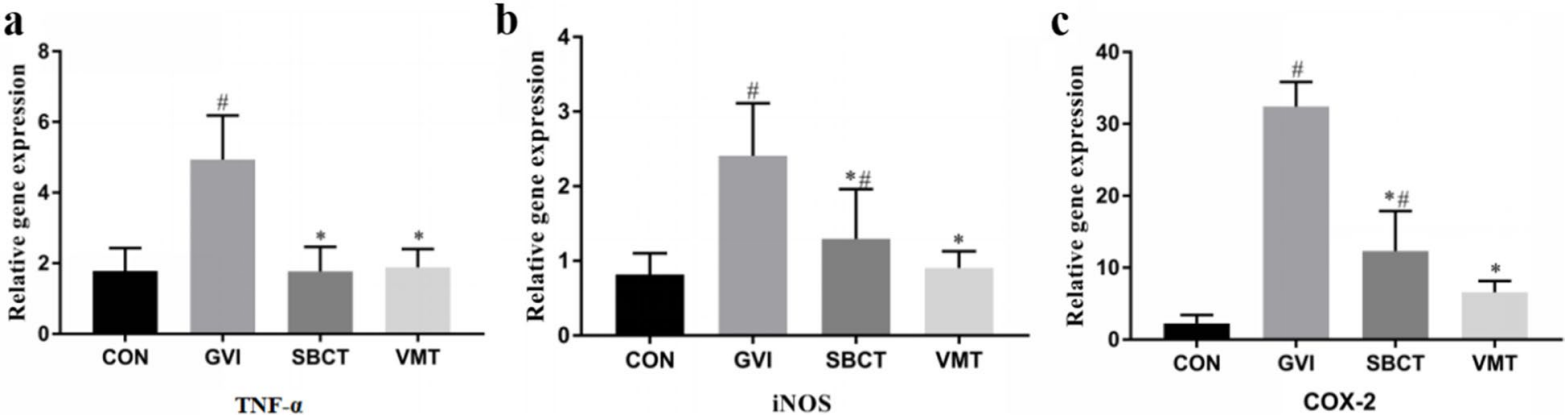
Expression of TNF-α, iNOS and COX-2 genes in the vaginal tissues of mice. Data are presented as the means ± standard error of mean obtained from three independent experiments. **a** TNF-α, tumor necrosis factor-α. **b** iNOS, inducible nitric oxide synthase. **c** COX-2, cyclooxygenase 2. #*P* < 0.05 vs. normal control group; *P < 0.05 vs. GVI group

To determine whether the innate immune system is involved in the anti-BV mechanism of SBCT and VMT. We investigated the effect of microbiota on NF-κB activation and inducible nitric oxide synthase (iNOS) and cyclooxygenase 2 (COX-2) expressions in vaginal tissues [25]. TNF-α is an important expressed gene for the NF-κB downstream pathway activation; thus it is used as a judgment marker for NF-κB activation. Two methods were used to treat *G. vaginalis*-induced BV mice, namely synthetic bacterial consortia and vaginal microbiota transplantation, respectively, for 2 weeks. According to the results of RT-PCR, TNF-α, iNOS, and COX-2 gene expressions were significantly increased in the vaginal tissues of *G. vaginalis*-induced BV mice. However, TNF-α, iNOS, and COX-2 gene expression were decreased in the two treatment groups (Fig. 4). To conclude, after probiotic treatment, the *G. vaginalis*-induced BV mice were able to inhibit the NF-κB activation, iNOS, and COX-2 effectively.

### Effect of SBCT and VMT on transcription factor expression of helper T cells in vaginal tissues

**Fig. 5:**
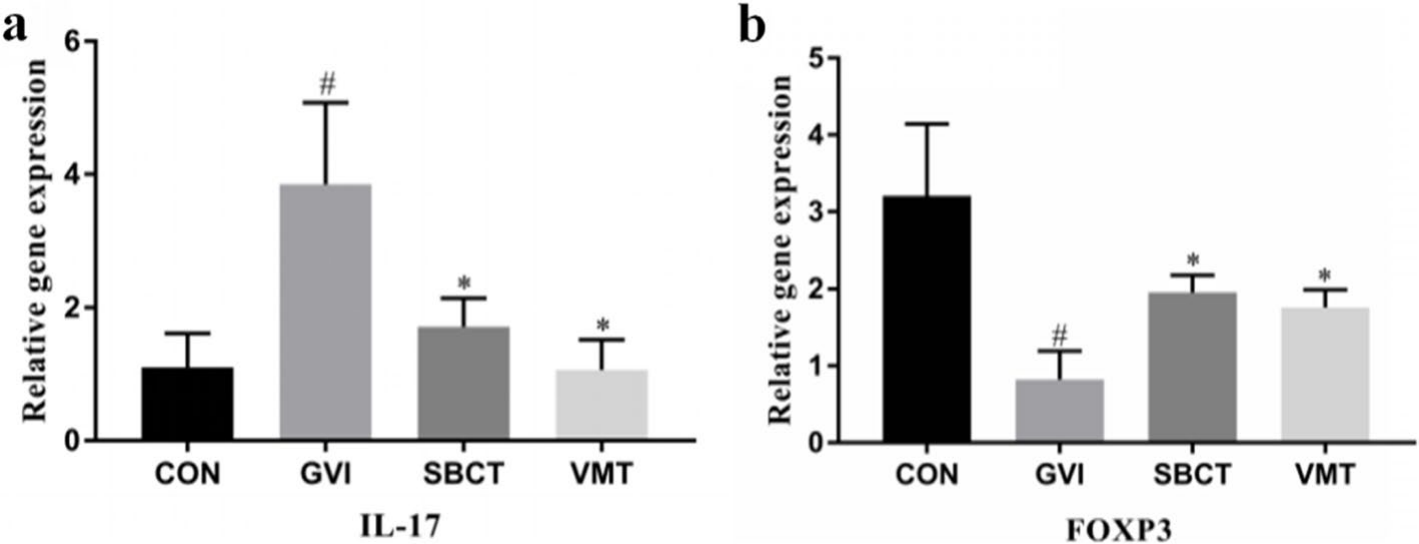
Expression of helper T cell transcription factors in the vaginas of mice. **a** IL-17, interleukin-17. **b** FOXP3, Forkhead Box Protein P3. Data are presented as the means ± standard error of the mean of three independent experiments. #*P* < 0.05 vs. normal control group; *P < 0.05 vs. GVI group

Next, we investigated the effect of vaginal microbiota transplantation on the transcription factor expression of helper T cells in mice. Interleukin-17 (IL-17) is a major effector secreted by T cells that regulates the release of pro-inflammatory cytokines and mediates NF-κB activation or inhibition. As shown in Fig. 5, *G. vaginalis*-induced BV mice displayed significantly upregulated IL-17 expression, and the SBCT and VMT groups displayed downregulated IL-17 expression. The qPCR showed that *G. vaginalis*-induced BV mice suppressed the expression of the Treg transcription factor Foxp3 [16, 45]. In addition, synthetic bacterial consortia or vaginal microbiota transplanted treated mice up-regulated BV-suppressed Forkhead Box Protein P3 (Foxp3) expression. Foxp3 is a key transcriptional regulator of Treg cells and forms a co-operative complex with nuclear factors of activated T cells, leading to the upregulation of Treg repressor genes. It has been shown that Foxp3 and Treg inhibition capacity are positively correlated. Overall, synthetic bacterial consortia or vaginal microbiota transplantation treatment is effective for treating *G. vaginalis*-induced BV.

### Vaginal microbiota analysis

**Fig. 6:**
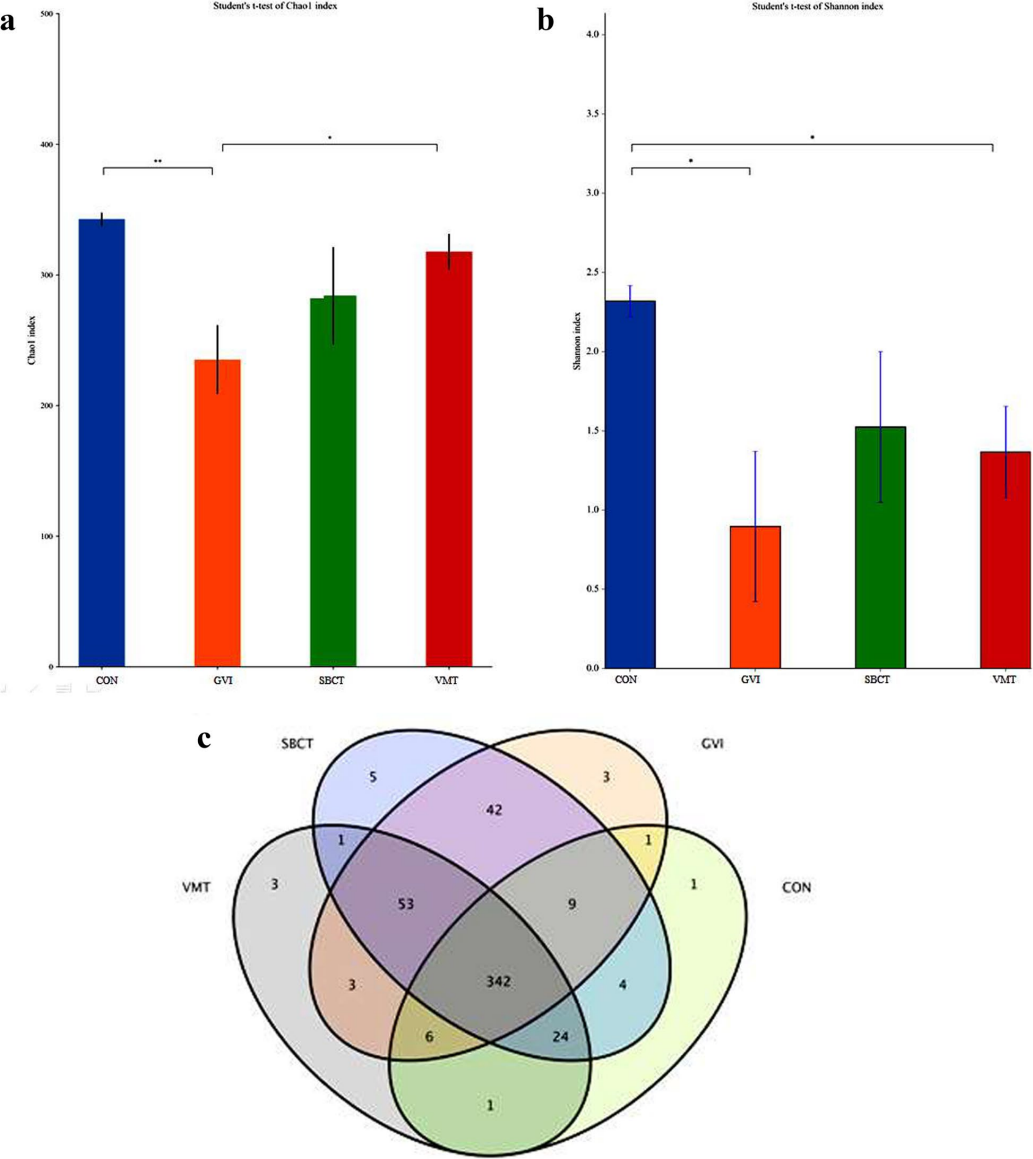
Evaluation of the vaginal microbiota in mice with vaginal microbiota dysbiosis using high-throughput sequencing: **a** Chao 1 index; **b** Shannon index; **c** Scalar Venn representation

There was a difference in the levels of microbial diversity between the groups. The trend representing the average Chao1 index was relatively high in the treatment of two groups and the CON group, compared to the lower abundance estimates in GV-inoculated mice (Fig. 6). We applied the Shannon index on unbinned data and found that SBCT or VMT significantly varied with that of the microbial diversity in the GVI group. The effective reads in the samples at a 97.0% similarity level were clustered and OTUs were obtained, with a total of 498 operational taxonomic units. As shown in Fig. 6c, a total of 342 common characteristic microorganisms were found in the four groups. The SBCT group had more, by number, of OUT than the GVI group, while the VMT group had fewer. In the vagina, microbial diversity and richness increased with treatment, which suggested that the vaginal microbiome underwent alterations.

**Fig. 7:**
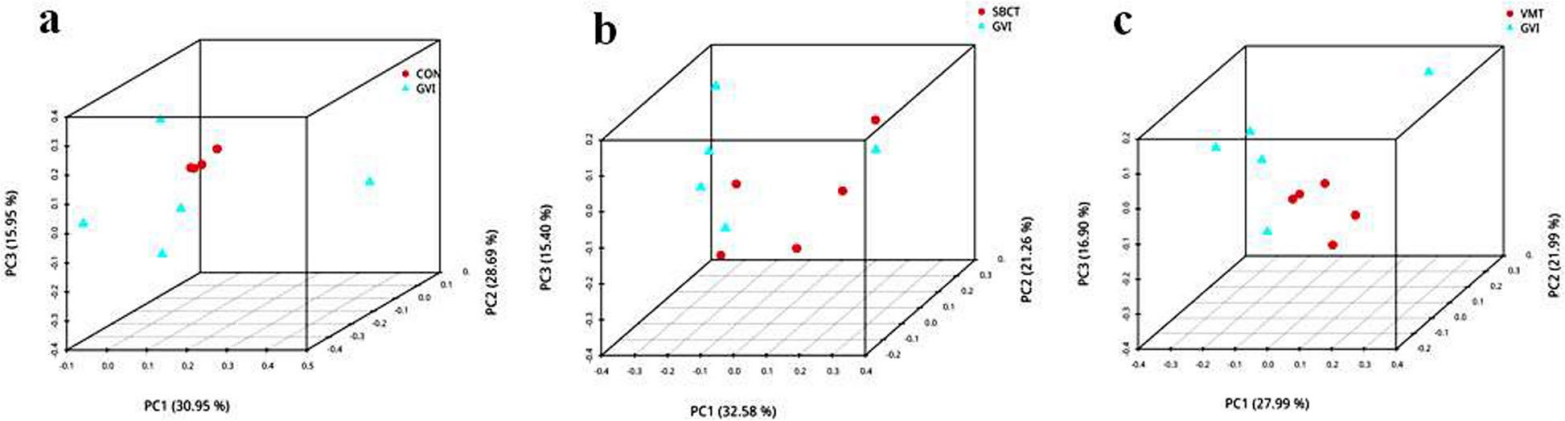
Principal coordinates analysis (PCoA) of the dissimilarity between the microbial samples: **a** CON and GVI group; **b** SBCT and GVI group; **c** VMT and GVI group

Next, we conducted a principal coordinate analysis (PCOA) based on unweighted UniFrac distance to measure the different groups’ related changes in microbial communities and their clustering relationships. It can be observed from the distribution of vaginal samples that the core regions of the three groups (CON, SBCT, and VMT) were relatively close to each other. Especially in the CON group and VMT group, the core regions almost overlapped and the SBCT group was partially clustered. In contrast, the GVI group had a large spatial displacement in the core regions and gradually moved the border regions (Fig. 7). The densely distributed area of post-treatment overlapped with the CON group in PCoA, indicating a more similar community composition.

**Fig. 8:**
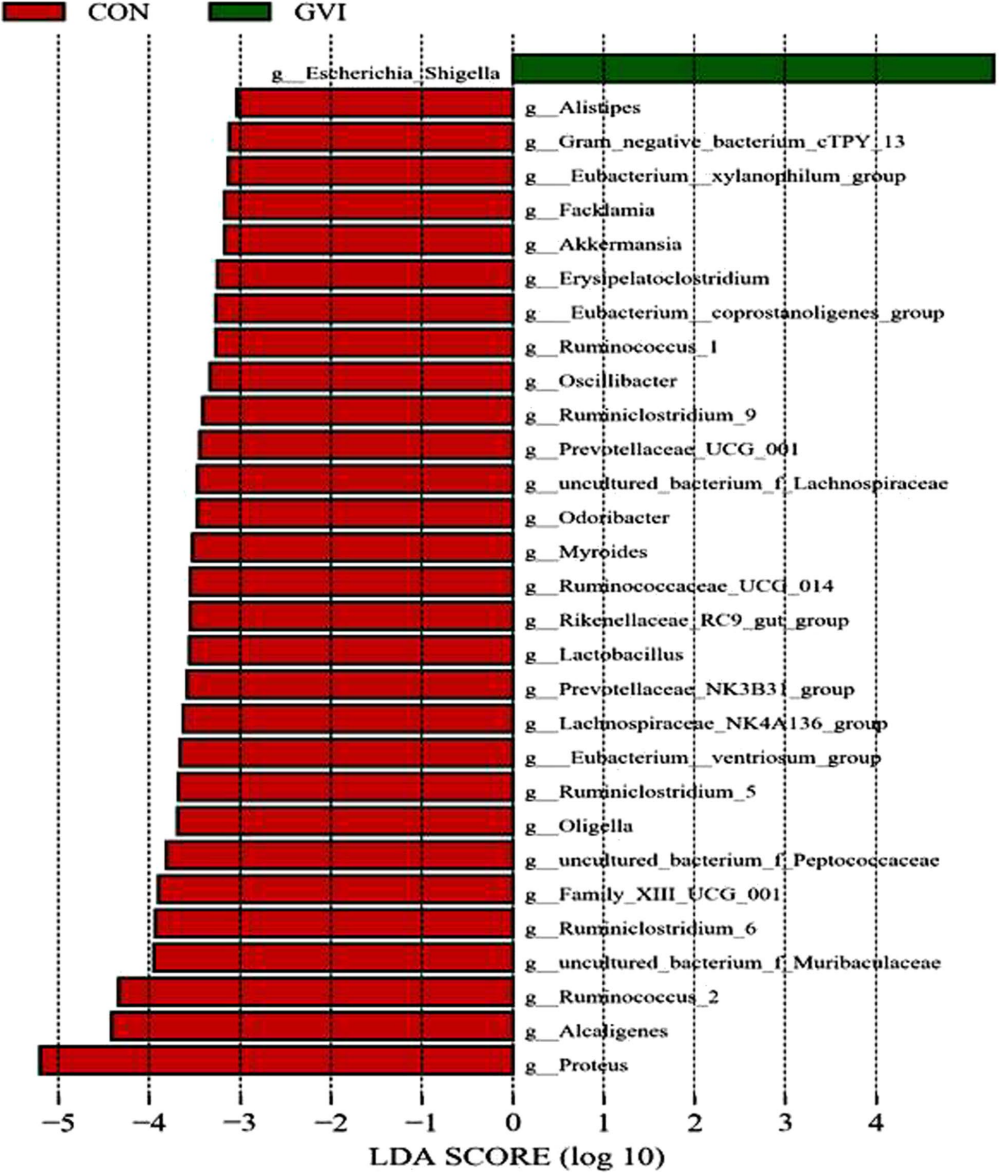
Comparison of the relative abundance at genus level by LEfSe analysis: **a** CON and GVI groups; **b** SBCT and GVI groups; **c** VMT and GVI groups

**Fig. 9:**
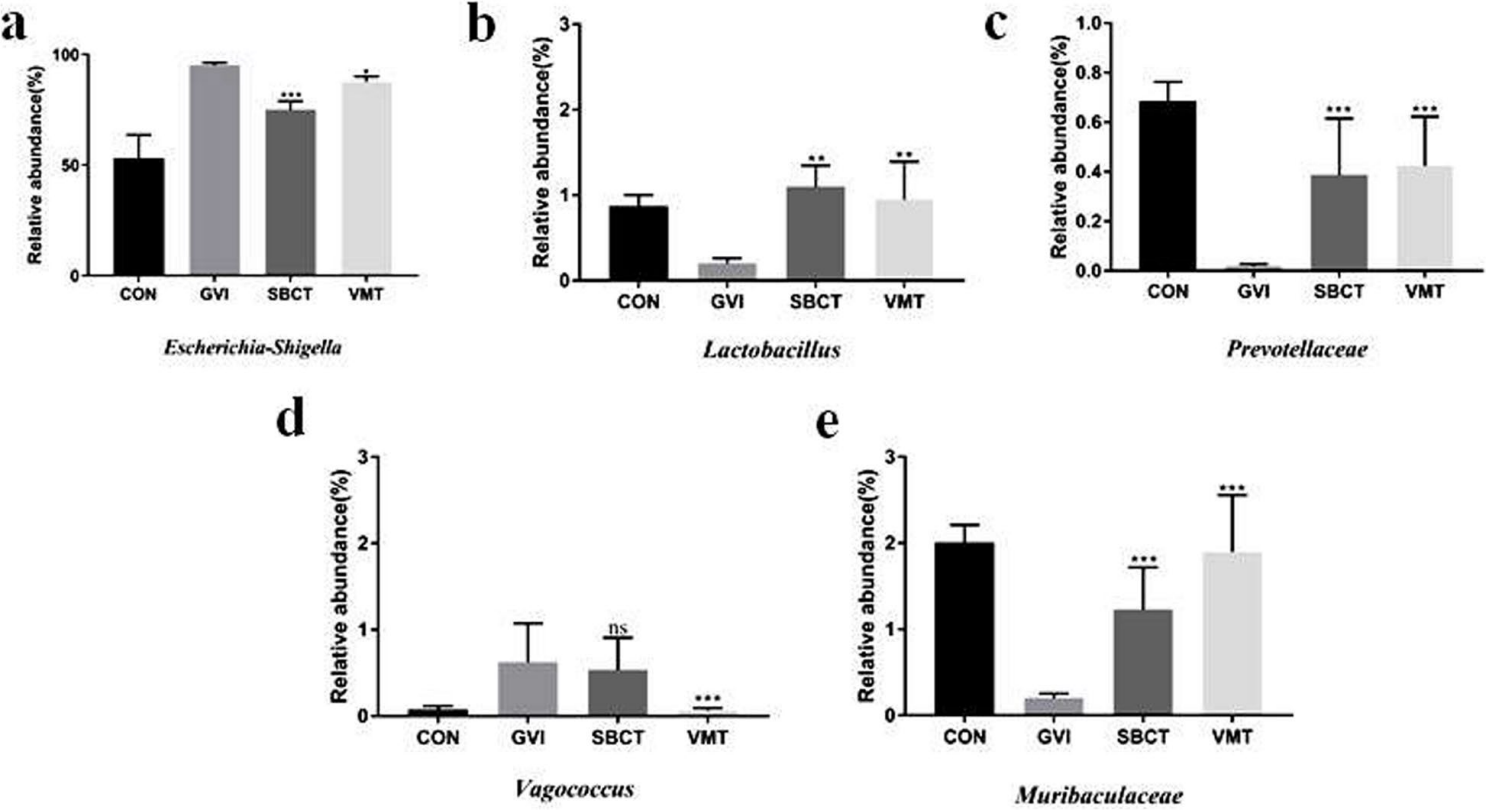
The relative abundance of the dominant bacteria of the vaginal microbiome at the genus level. *P < 0.05 vs. GVI group

To further illuminate these differences in microbial diversity and community structure, we analyzed the relative abundance of the dominant bacteria. The GVI mice have higher amounts of Proteobacteria and fewer Firmicutes and Bacteroides, while the dominant bacterial phyla in the vaginal microbiomes were identical. This post-treatment compositional change was mostly dominated by higher amounts of *Lactobacillus*, combined with fewer amounts of the *Escherichia-Shigella*. Other genera, including *Muribaculaceae*, *Prevotellaceae*, and *Anaerolineaceae*, were increased upon successfully induced remission of BV (Fig. 8). The increases of various *Lactobacillus*, which are beneficial to the vagina of healthy, maintained the low pH of the vaginal environment and maintained the microecology in a relatively balanced state. The vaginal microbiome was accompanied by an increase in the amount of *Lactobacillus* and a decrease in the amount of *Escherichia-Shigella* and *Vagococcus* (Fig. 9). These variations may account for the alteration in the microbial diversities in SBCT or VMT group, also leading to partial similarities in the community structures of the two groups. After treatment, the vagina of mice harbored more bacterial taxa with high abundance, evenness, and interindividual similarities. The data results demonstrated that the SBCT or VMT could regulate the vaginal microbiome disorder and thus be an effective treatment of BV.

**Fig. 10:**
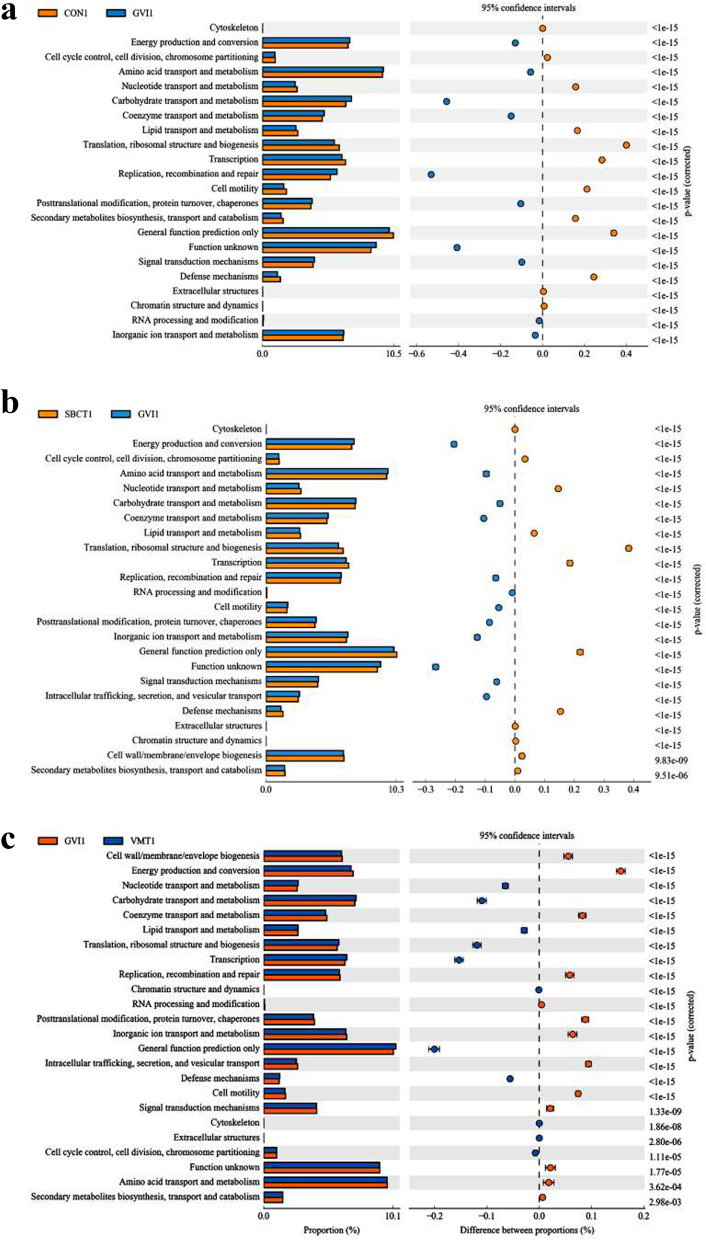
Clusters of orthologous groups (COG)-predicted functional classification with different treatments. **a** CON and GVI groups. **b** SBCT and GVI groups;. **c** VMT and GVI groups

Functional microbiome changes after the post-treatment, as evaluated using the Clusters of Orthologous Genes (COG), revealed different functional clusters that separated the BV microbiome from the CON group. Analysis of sequencing data showed that the abundance of genes responsible for seven pathways was upregulated in both SBCT and VMT groups, such as chromosome partitioning, nucleotide transport and metabolism, lipid transport and metabolism, translation, ribosomal structure and biogenesis, transcription, general function prediction, and defense mechanisms. And down-regulated in the abundance of genes involved in eight pathways compared to the GVI group, such as energy production and conversion, coenzyme transport and metabolism, replication, recombination and repair, post-translational modification, protein turnover, chaperones, inorganic ion transport and metabolism, cell motility, signal transduction metabolism, and intracellular trafficking, secretion and vesicular transport (Fig. 10). Changes in the functional potential of the microbiota were transformed after treatment, which facilitated host inhibition of the growth of harmful bacteria and modulation of the immune response.

## Discussion

Female vaginal microbial communities are less diversified than those of the gastrointestinal tract and are dynamically changed during the female menstrual cycle. The vaginal microbiota ecosystem at the edge between immune homeostasis and inflammation has been recognized as playing an important role in microbial-host interactions [44]. The vagina of healthy women contains *Lactobacillus*, which produces metabolites such as lactic acid and hydrogen peroxide and maintains a healthy vaginal ecosystem that resists pathogenic infections. It is important to note that *Lactobacillus* does not dominate the composition of the vaginal microbiota in a few women. In the woman with bacterial vaginosis, the *Lactobacilli* was replaced by anaerobic bacteria such as *Gardnerella vaginalis*, *Atopobium vaginae*, *Ureaplasma urealyticum*, *Mycoplasma hominis*, and others [1]. Maintenance antimicrobial treatment is often suggested, but it can be prone to vaginal candidiasis and resistant infections. Although VMT is in its early stages, VMT is expected to be the potential approach to treating vaginal ecological disorders [23, 28, 40], but similar to fecal microbiota transplantation (FMT), difficulties such as the unrecognized transmission of infectious diseases and antibiotic resistance genes still pose significant concerns regarding the acceptability and safety of VMT.

In this study, we successfully introduced *G. vaginalis* into the vagina of mice to cause vaginal ecological disorders for the development of a model of BV. SBCT and VMT were used to treat GV-inoculated mice and alleviated the inflammation of vaginal infection. We found that VMT was more effective than SBCT in suppressing GV-induced inflammation. Perhaps because VMT is introduced into the vagina as a whole system, allowing the microbiota to maintain better stability, and VMT contains bacteria that are not present in the synthetic preparation. However, the bacterial strains we selected for SBCT were able to produce natural antibacterial products such as hydrogen peroxide and lactic acid to resist pathogenic bacteria [30]. Previous studies reported that oral or vaginal administration of a *Lactobacilli* mixture could attenuate GV-inoculated mice by regulating the innate and adaptive immune responses [20]. Other reports also displayed that probiotics can inhibit the growth of *G. vaginalis* in vitro [39]. Lactic acid may also directly affect host tissues by modulating the immune system and gene expression [49]. In the present study, the administration of SCBT and VMT reduced the number of *G. vaginalis* detected in the vagina. Simultaneously, we verified with H&E staining that SCBT could restore the vaginal epithelial cell damage to some extent. After 2 weeks of treatment, the thinning of vaginal epithelial cells and cellular infiltration were significantly improved, and vaginal epithelial cells recovered better in VMT mice than in the SBCT group. The ELISA and qPCR results showed that SCBT and VMT inhibited GV-induced inflammatory cytokines in vaginal tissues and serum. Treatment with SCBT and VMT also inhibited IL-1β and IL-8 expression in GV-infected mice. In contrast, IL-10 expression was increased in SCBT and VMT mice. Decreased expression of pro-inflammatory cytokines and increased expression of anti-inflammatory cytokines in SCBT and VMT mice were found. Similar results have been previously reported; VMT or probiotic combination is a potential therapy for the treatment of GV infection [10, 29, 33]. Our results suggest that SBCT and VMT may alleviate the inflammatory response by inhibiting the production of pro-inflammatory cytokines.

NF-κB is a key transcription factor that increases the expression of pro-inflammatory enzymes and cytokines [27]. SBCT and VMT suppressed TNF-α and IL-17 expression in vaginal tissues, blocked inflammatory factors from binding to TLR4, and inhibited NF-κB activation. It is well-known that overproduction of nitric oxide (NO) by iNOS is associated with various pathophysiological processes, including inflammation. iNOS could be used as a marker and a treatment instrument in diagnostic and inflammatory processes [24]. In particular, COX-2 promotes apoptotic resistance, proliferation, angiogenesis, inflammation, and invasion, and is a major downstream regulator of NF-κB in cells [17]. The results showed that SBCT and VMT significantly suppressed TNF-α, iNOS and COX-2 expression. The administration of SBCT and VMT could inhibit NF-κB activation, iNOS, and COX-2 expression, and confirmed the involvement of the innate immune system in the anti-BV mechanism of the microecological environment. SBCT or VMT more potently inhibited the expression of FOXP3 and IL-17 in GV infection, which are involved in adaptive immunity. Meanwhile, the anti-BV effect of VMT might be more effective than the SBCT.

Next, we focused our investigation on whether SBCT and VMT change microbiome diversity and structure and change the possible relationship between microbiome change and their predicted function. To provide basic information to enhance our understanding of the enhancement mechanisms of microbiome changes. As described by the alpha-diversity analysis, the overall diversity was influenced by the GVI and other groups. Meanwhile, the ß-diversity analysis showed that overall community structure was significantly different between the groups. In addition, administration of the SBCT and VMT in GV-infected mice restored microbial diversity to normal levels (CON group). Experimental data showed that the composition of the vaginal microbiota consists mainly of Proteobacteria, Firmicutes, Bacteroidetes, and Acidobacteria at the phylum level. We have demonstrated that VMT and SCBT increased the abundance of *Lactobacillus* significantly and reduced the overgrowth of *Escherichia-Shigella* and *Vagococcus*. *Lactobacillus* spp. proves to be the most important bacteria in women's vaginas. The production of lactic acid and hydrogen peroxide by these strains at pH (pH4.0–4.5) is beneficial to women, thus maintaining an acidic and healthy microenvironment. More recent work has highlighted that populations of *Lactobacillus* are typically not comprised of a single strain and display a substantial amount of intraspecies diversity. This intraspecific diversity might be a key determinant of community stability because it buffers the dominant *Lactobacillus* population against perturbations [13, 36, 38]. Recent experimental studies have shown that four out of five recipients who received VMT achieved long-term remission [29]. This study could then be translated to traditional *Lactobacillus* probiotic formulations with increased safety.

COG pathways provided a comprehensive overview of a large number of functional groups between the BV- and non-BV-associated samples. COG analysis showed that VMT up-regulated genes are the responsible pathways in chromosome partitioning, nucleotide transport and metabolism, lipid transport and metabolism, translation, ribosomal structure and biogenesis, transcription, general function prediction, and defense mechanisms. And downregulated genes involved in energy production and conversion, coenzyme transport and metabolism, replication, recombination and repair, post-translational modification, protein turnover, chaperones, inorganic ion transport and metabolism, cell motility, signal transduction metabolism, intracellular trafficking, secretion, and vesicular transport. These results suggest that VMT and SBCT might be achieved by modulating the host’s vaginal microbiota composition in order to alleviate bacterial vaginitis. Cho et al. discovered that the active gene expression in the BV-associated samples suggests that the consortium of microbiota promoted the gene expression machinery to develop BV in the subject [8]. Simply, the large difference in the number of COGs between BV and non-BV samples could be determined by the number of microorganisms actively living in the vagina.

Indeed, VMT is potential therapy in the clinical treatment of bacterial vaginosis, showing a significant improvement in symptoms, Amsel criteria, and the re-establishment of the vaginal microbiome [29], but the safety and reliability of VMT in treatment need to be further analyzed and studied. Here, we have verified the therapeutic potential of SBCT in vaginal dysbiosis in mice and the potential mechanisms for treating vaginal dysbiosis and restoring the vaginal microbiome to normal levels. However, our study did not have sufficient power to test whether VMT and synthetic bacterial consortia are effective. Further study with larger sample sizes is also needed, especially the potential mechanisms and a clinical experiment to validate them. Moreover, the acceptability and safety of VMT and synthetic bacterial consortia in clinical trials need to be further investigated. In conclusion, synthetic bacterial consortia transplantation and vaginal microbiota transplantation are effective in modulating innate and adaptive immune responses against *G. vaginalis* infection and maintaining the balance of vaginal microbiota by enhancing probiotic growth and suppressing the growth of pathogens in mice, but they should be tested in human for further study of the effects of SBCT and VMT in the treatment of BV.

## Materials and methods

### Cultivation of *Lactobacillus* strains

The lactic acid bacteria strains for synthetic bacterial consortia were isolated from the vaginal discharge of five healthy women. Each participant provided informed consent, and the research was approved by the institutional ethics committee of Lishui People’s Hospital (Ethics Number: 2021-241). Isolates of *L. crispatus*, *L. rhamnosus*, *L. salivarius*, and *L. plantarum* were selected for synthetic bacterial consortia construction. These strains were cultured under aerobic conditions in de Man, Rogosa, and Sharpe (MRS) broth at 37 °C for up to 24 h. The bacteria cultures were harvested and suspended in phosphate buffer solution (PBS; pH 7.0) at a density of 3 × 10^9^ CFU mL^-1^ [46]. Vaginal microbiota transplanted discharge from healthy female mice was diluted with PBS at a cell density of 3 × 10^8^ CFU mL^−1^ for vaginal treatments.

*Gardnerella vaginalis* ATCC 14018 (GV) was cultured in Columbia Blood Agar Base Medium and incubated under anaerobic conditions using sealed anaerobic jars at 37 °C for up to 36 h. The fermentation broth was harvested and suspended in PBS at a density of 3 × 10^9^ CFU mL^−1^ for vaginal injection.

### Ethics statement

The procedures involving the animals and their care were conducted in conformity with national and international laws and policies. The animal experiments were approved by the Laboratory Animal Care and Use Committee of China Pharmaceutical University.

### Animals and treatment

Female BALB/c mice obtained from Charles River (Beijing, China) were used at 5 weeks of age. The mice were fed a standard diet and a standard amount of water. The mice were kept in wire cages at climate-controlled conditions (at 50 ± 10% humidity and 24 °C) for 1 week prior to starting the formal experiment procedure. During the model stage, 3 days prior to GV treatment, mice were induced into a pseudoestrus condition by intraperitoneal injection of 0.3 mg of β-estradiol-3-benzoate in 100 μL of olive oil [26]. Twenty-eight mice were divided into four groups randomly: Control group (CON), GV-induced BV group (GVI), GV-induced BV treated with synthetic bacterial consortia transplantation group (SBCT), GV-induced BV treated with vaginal microbiota transplantation group (VMT). Mice in GVI, SBCT and VMT were anesthetized with 2.5–3.5 v/v of isoflurane gas and then inoculated with 3 × 10^9^ CFU mL^−1^*G. vaginalis* in 20 μL of cell suspensions, once at 24 h intervals, bacterial suspension was inoculated via a mechanical suction tube into the vaginal cavity, near the cervix [11, 22, 37]. The control group (CON) was treated with saline instead of the GV suspension. After being induced with GV, the bacterial burden was monitored by the flat colony counting method every 2 days.

**Fig. 11:**
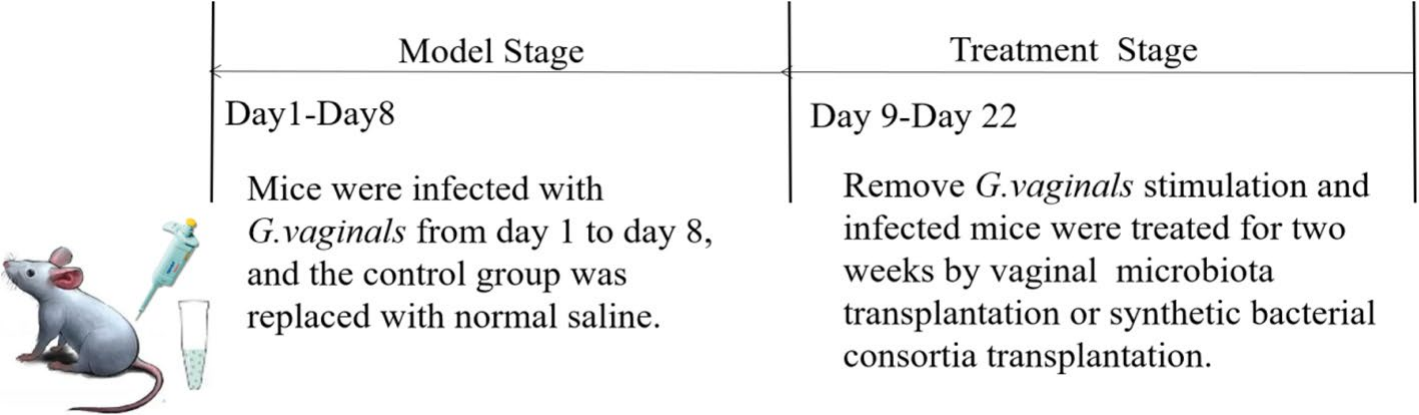
Experimental scheme for the treatment of vaginal dysbiosis by synthetic bacterial consortia transplantation and vaginal microbiota transplantation

The GV-induced BV mice were treated with synthetic bacterial consortia or vaginal microbiota transplantation, respectively every day. For the preparation of probiotic samples, 20 μL of the suspension of synthetic bacterial consortia adjusted to 3×10^9^ CFU mL^-1^ in PBS was vaginally inoculated; 3 × 10^8^ CFU mL^−1^ vaginal microbiota transplant suspension was vaginally inoculated. The GV-infected control group (GVI) was treated with saline instead of synthetic bacterial consortia or vaginal microbiota with successive doses of treatment for 14 days (Fig. 11). After 14 days of treatment, the mice were euthanized, and the vaginas were removed and washed with 200 μL of PBS. After washing, the vaginas were stored at – 80 °C and used for mRNA extraction or histological inspection. Mice vaginal discharges were used for bacterial DNA extraction and high-throughput sequencing analysis.

### Histopathological examination

The effect of SBCT and VMT in mice infected with *G. vaginalis* was assessed by analyzing histopathological changes in vaginal tissues. Vaginal tissues were fixed in a 10% neutral buffered formalin solution for 72 h and embedded in paraffin. The samples were stained by hematoxylin and eosin (H&E) and evaluated histopathological change compared with CON and GVI groups.

### ELISA for cytokines detection

Mice were euthanized at the end of the treatment and blood samples were collected. Serum was prepared by centrifugation at 3000×*g* for 10 min at 4 °C. The concentration of cytokines including interleukin-1β (IL-1β), interleukin-8 (IL-8), and interleukin-10 (IL-10) in the serum were measured using the corresponding commercial kits (Sino Best Biological Technology Co., Ltd., Shanghai, China) according to the manufacturer’s protocol. Samples were diluted in an assay buffer to adjust the concentration to the linear range of the standard curve and the serum from CON group were used as control.

### Quantitative polymerase chain reaction (qPCR)

Total RNA was isolated from vaginal tissue using TRIzol® reagent (Vazyme, Nanjing, China) in accordance with the manufacturer’s protocol. The RNA concentration was then measured and quantified. For each sample, 1.00 μg of total RNA was reverse transcribed for complementary DNA (cDNA) synthesis using a PrimeScriptTM RT Reagent Kit with a gDNA Eraser (Vazyme, Nanjing, China). Subsequently, real-time PCR analysis was performed for TNF-α, IL-17, Foxp3, and glyceraldehyde-3-phosphate dehydrogenase (GAPDH) [31] (Table 1), samples from rom CON group were used as control. RT-qPCR reactions were performed using Power SYBR green PCR master mix and the thermal cycling conditions were as follows: 42 °C for 5 min, 95 °C for 10 s, followed by 40 cycles of denaturation, followed by amplification at 95 °C for 5 s and 60 °C for 30 s. Changes in gene levels relative to GAPDH were recorded and calculated using Microsoft Excel.

**Table 1:** Primers used in this study

Gene	Primer sequence
TNF-α	forward: 5′-TCTTCTCATTCCTGCTTGTGG-3′
	reverse: 5′-GGTCTGGGGCATAGAACTGA-3′
iNOS	forward: 5′-CCTTGCACTGCCAAGAATTTG-3′
	Reverse: 5′-CATTGCGTCACTGGATAGTAGTT-3′
COX-2	Forward: 5′-AAAGTTCAGCCATTGTACAGCAGG-3′
	Reverse: 5′-GAATCTCCTAGAACTGACTGG-3′
IL-17	Forward: 5′-TTTAACTCCCTTGGCGCA AAA-3′
	reverse 5′-CTTTCCCTCCGCATTGACAC-3′
Foxp3	Forward: 5′-CCCATCCCCAGGAGTCTT-3′
	Reverse: 5′-ACCATGACTAGGGGCACTGTA-3′
GAPDH	Forward: 5′-TGAGTGGCAAAGTGGAGAT-3′
	Reverse: 5′-TTTGCCGTGAGTGGAGTCAT-3′

### Vaginal microbiota analysis

The extracted genomic DNA was used as a model to amplify the V3-V4 region of the 16 S rRNA genes using the universal primer 338F/806R (5′-ACTCCTACGGGAGGCAGCAG-3′, 806R 5′-GGACTACGVGGGTWTCTAAT-3′). PCR augmentation was operated in an entire volume of 50 μL, which consisted of 0.2 μL Q5 High-Fidelity DNA Polymerase, 10 μL Bμffer, 10 μM of each primer, 10 μL High GC Enhancer, 1 μL dNTP, and 50 μL genome DNA. Thermal cycling conditions were as follows: an initial denaturation for 5 min at 95 °C, followed by 15 cycles for 1 min at 95 °C, 50 °C for 1 min and 72 °C for 1 min, with a final extension for 7 min at 72 °C. A second round of PCR was then operated in a 40-μL reaction which consisted of 8 μL dd H2O, 20 μL 2 × Phμsion HF MM, 10 μM of each primer and 10 μL PCR products from the first step. Thermal cycling conditions were as follows: an initial denaturation for 30 s at 98 °C, followed by 10 cycles for 10 s at 98 °C, 65 °C for 30 s and 72 °C for 30 s, with a final extension for 5 min at 72 °C. Finally, total PCR products were quantified by Quant-iT™ dsDNA HS Reagent and put together. High-throughput sequencing analysis for bacterial rRNA genes was operated on the purified, pooled sample by using the Illumina Hiseq 2500 platform (2 × 250 paired ends) at Biomarker Technologies Corporation (Beijing, China). All bioinformatics data were analyzed on the free online platform of BMK Cloud Platform and the GenBank accession number is as follows: PRJNA814478.

### Statistical analysis

All experimental results data were evaluated by mean ± standard error of the mean. Statistical significance was then analyzed using one-way ANOVA and finally using GraphPad Prism software and unpaired *t* tests. If data were not normally distributed, then the nonparametric Kruskal-Wallis test was used, and pairwise comparison was done using Dunn’s multiple comparison tests; *p* < 0.05.

## Data Availability

The raw data for the 16S rRNA gene sequence has been deposited in the NCBI BioProject database (https://www.ncbi.nlm.nih.gov/bioproject/) under accession number: PRJNA814478. The other data of this study are available on request from W.C.
